# Individual and context-evoked antecedents of exploration-exploitation performance

**DOI:** 10.3389/fpsyg.2023.1167135

**Published:** 2023-12-22

**Authors:** Jan Richner, Zorica Zagorac-Uremović, Daniella Laureiro-Martínez

**Affiliations:** Chair of Technology and Innovation Management, Department of Management, Technology and Economics, ETH Zürich, Zürich, Switzerland

**Keywords:** exploration and exploitation, cognitive flexibility, executive functions, emotions, leadership, Carnegie, microfoundations

## Abstract

A central issue within the Carnegie approach is the exploration-exploitation tension that lies behind organizational adaptation. After decades of research, there is still little understanding of how the combination of individual and context-evoked differences affects exploration-exploitation performance. To address that issue, we build on recent psychological and neuroscientific studies to develop and test an integrative model. The model considers two individual antecedents (personality and cognitive flexibility) and three context-evoked antecedents that take place along different time horizons (recent stress, present emotional states, and present task motivation). We rely on a lab-in-the-field study of 282 leaders within the Swiss Armed Forces—an organization that exhibits the exploration-exploitation tension in an accentuated form. Using structural equation modeling, we conduct a multiple-mediation path analysis aimed at testing complex interactions between multiple variables. Our findings highlight the need to take an integrative approach; cognitive flexibility mediates the positive effect of the personality trait of emotional stability on exploration-exploitation performance, however, both cognitive flexibility and emotional stability play unique, underlying roles in explaining how organizational leaders interpret the context. Emotional stability decreases the negative effect of recent stress on a leader’s cognitive flexibility. Cognitive flexibility, in turn, mediates the effect of the present positive affective signals of task motivation on exploration-exploitation performance. These findings shed new light on our understanding of how adaptive leaders leverage positive and negative context-evoked antecedents that, in turn, affect cognitive flexibility and exploration-exploitation.

## Introduction

The Carnegie approach places a strong emphasis on the study of issues that affect daily organizational life. Among those, a central issue relates to the fundamental tension that lies behind adaptive behavior and emerges when making decisions that balance exploratory and exploitative behaviors. Organizations must constantly explore new options, but they often fail as they focus on exploiting known options to sustain efficiency and fall prey to organizational inertia (Tripsas and Gavetti, 2000). This is particularly likely when organizations are currently experiencing success—something the Carnegie approach has studied as the myopia of learning (Levinthal and March, 1993)—or when they operate in regulated ways following standard operating procedures that bring short-term efficiency advantages at the expense of flexibility (Cyert and March, 1963). To overcome myopia and rigidity, organizations must rely on leaders who manage exploration and exploitation dynamically by making adaptive decisions appropriate to a given context at any moment in time. While the Carnegie approach emphasizes the importance of both context and individual differences in human behavior (e.g., Simon, 1997; O’Reilly and Tushman, 2004), a model that integrates the combined effects of different antecedents on exploration-exploitation performance is lacking (Gavetti et al., 2007). Our goal is to combine psychology and neurosciences (e.g., Lerner et al., 2015; Pessoa, 2017) to propose and test a model that studies the interplay between fundamental psychological categories that are considered antecedents of human behavior: individual antecedents (categorized under personality traits and cognitive flexibility) and context-evoked antecedents (categorized as affective signals pertaining to different time horizons such as recent stress, present emotional states, and task motivation). We contribute to the Carnegie approach by revealing how individual and context-evoked antecedents influence each other and, in turn, jointly affect exploration-exploitation performance.

We build on Herbert Simon’s (1967, 1997) and March and Simon’s (1958, 2004) ideas that emotions and motivations (i.e., affective signals) are central antecedents at the intersection between organizational context and individual organizational leaders. Affective signals describe how environmental signals evoke individual responses by attracting their attention through a “complex interweaving of affective and cognitive processes” (March and Simon, 1958, 2004, p. 151). In line with the state-of-the-art understanding of affective signals (e.g., Zadra and Clore, 2011), we propose that the organizational context is likely to indirectly affect leaders’ exploration-exploitation performance through the context-evoked antecedents of recent situational stress, present emotional states, and task motivation. While these context-evoked affective signals reflect how the context influences leaders, the perceived intensity of these affective signals can vary from individual to individual (e.g., Sherman et al., 2012), which, in turn, might have an effect on exploration-exploitation performance.

**Figure 1:**
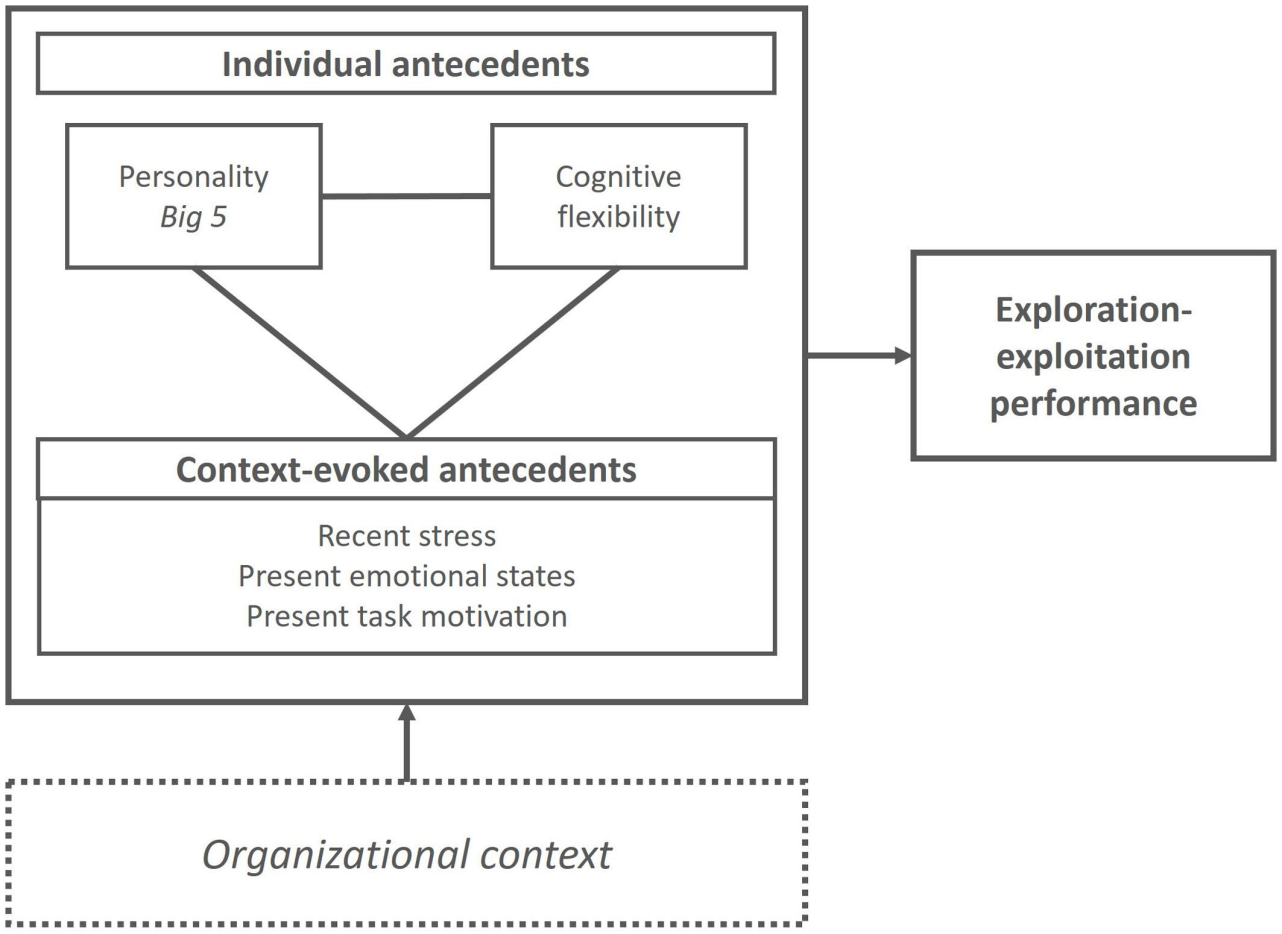
Basic conceptual framework.

We investigate the interplay between individual antecedents (i.e., personality traits and cognitive flexibility) and context-evoked antecedents (i.e., recent stress, present emotional states, and task motivation). Figure 1 provides an overview of the antecedents of exploration-exploitation that we study at different levels (individual and context-evoked) and the interactions between them (e.g., how the organizational context evokes some antecedents that interact with individual antecedents). The theory section will develop arguments for each of the variables and outline the directionality of the different interactions between them.

The study of such interactions requires a method that will allow for controlled measurements in the field and an analysis of multiple interactions. Thus, we rely on a lab-in-the-field study and collect rich individual and context-evoked data from a sample of 282 leaders training and practicing leadership skills within the Swiss Armed Forces. We analyze the data by means of structural equation modeling, which allows us to examine complex interactions between individual and context-evoked antecedents (Zyphur et al., 2023) in an organization often characterized by rigidity. In fact, the Swiss Armed Forces exhibit the tension between exploration-exploitation in an accentuated form: the organization is large and heavily regulated and, at the same time, must continuously prepare to operate under unknown conditions. To test our model, we draw parallels between typical war simulation exercises and a task paradigm that captures the essence of dynamic exploration-exploitation decisions under conditions in which organizational leaders must make multiple, adaptive decisions over a period of time. Although war is a rare event, it is the *raison d’être* for military organizations and, therefore, forms the core of military leadership education programs (Hirst, 2022). In fact, most of everyday organizational life in the military centers on training that focuses precisely on the exploration-exploitation tension using a variety of war simulation exercises. Thanks to continuous training, organizational members learn the standard procedures that allow for efficiency and coordinated action while also practicing how to select, recombine, or redeploy some of those standard procedures in different ways should the context change and decisions need to be made under greater time pressure and/or resource scarcity (Cyert and March, 1963).

Our study identifies key antecedents that enable or hinder leaders’ ability to deal with exploration-exploitation decisions. First, we identified cognitive flexibility with its core components—vigilance, working memory, and switching—as a central individual antecedent behind exploration-exploitation. Cognitive flexibility not only positively and directly affects exploration-exploitation performance but also mediates the positive effect that the personality trait of emotional stability has on it. Second, we found that emotional stability mediates the negative effect of recent stress on cognitive flexibility. Thus, this personality antecedent plays an additional role as a protective shield for organizational leaders’ cognitive flexibility and allows them to cope with potentially negative context signals when they must make exploration-exploitation trade-off decisions. Third, and in line with both received theory (e.g., Dolan, 2002; Phelps et al., 2014; Lerner et al., 2015; Pessoa, 2017) and our model, we find that cognitive flexibility mediates the effect of present task motivation on exploration-exploitation performance. This finding indicates that contextual, affective signals can inform and interact with—and not just bias—cognition. Taken together, the results provide empirical evidence that leaders make exploration-exploitation decisions in a truly situated manner: they interact with the context by leveraging cognitive flexibility and specific personality antecedents to process helpful and potentially harmful contextual cues to achieve higher exploration-exploitation performance.

The current study’s findings contribute to our understanding of the antecedents of adaptive exploration-exploitation decisions in organizational leaders in two main ways. First, while not claiming causality, our study contributes to Carnegie literature by putting forward and testing a microfoundational model that studies the complex interactions of individual and context-evoked antecedents that affect exploration-exploitation performance. Second, by attending to both individual and context-evoked antecedents of behavior, our study “exports” to psychology an organizationally situated understanding of exploration-exploitation—a central tenet of the Carnegie approach—and proposes that, in addition to individual antecedents, it is important to study a category of variables that considers how individuals’ affective signals capture elements of the context over different time horizons. This is difficult in a pure lab study, but the lab-in-the-field approach of our study allows us to capture context-evoked antecedents. Specifically, we consider how the context affects the individual via three context-evoked variables: recent stress, present emotional state, and present task motivation. Additionally, the study allows us to draw parallels between empirical and practical task paradigms—here, between the four-armed bandit task and war simulation exercises, which are fundamental activities for leadership development in the context of our study (Augier et al., 2018). The stringent mode of aligning a lab task to an organizational task enables us to increase the external validity of constrained laboratory tasks without losing the advantages of the internal validity associated with these tasks.

## Adaptive exploration-exploitation decisions

The tension between exploration and exploitation is pervasive and involves issues that can take place in different timeframes and at different levels. At its core, it is a tension involving choices that “must be made between gaining new information about alternatives and thus improving future returns (which suggests allocating part of the investment to searching among uncertain alternatives) and using the information currently available to improve present returns (which suggests concentrating the investment on the apparently best alternative)” (March, 1994, p. 237). Exploration-exploitation choices are faced by everyone from entire armies at war (should a troop focus intensively on a known site or explore new battlegrounds?) to CEOs (should the company invest in its current market or explore new ones?).

Organizations must rely on their adaptive leaders’ ability to manage the exploration-exploitation tradeoff. An adaptive leader is one who can decide for themselves and for others, when to stick to a well-known option and when to try out an alternative one—i.e., when to stay and when to go (Laureiro-Martínez et al., 2015). Thus, the adaptive leader can identify when to switch between exploratory and exploitative behaviors; high exploration-exploitation performance is not achieved by switching between exploration and exploitation per se but by doing so at the right moment—for instance, in reference to the perceived level of uncertainty in a situation (Mehlhorn et al., 2015) or in response to performance feedback related to a preceding decision (Levinthal and March, 1993).

There is agreement in the management literature that “the ability to dynamically balance exploration and exploitation” (Luger et al., 2018, p. 450) provides an adaptive solution to the tension between the two and leads to better outcomes (O’Reilly and Tushman, 2004). However, not everyone has the same dynamic balancing ability (Raisch et al., 2009). Various studies have aimed to understand the antecedents that lead to appropriate switching between exploration and exploitation. Some studies have focused on individual variables, while others have focused on variables that capture particular aspects of the context. Simon (1997) proposed two main sets of mechanisms that affect behavior: those that are for the most part internal (“their situs is in the human mind”) and those that are “largely external to the individual, although they usually imply his [*sic*] sensitivity to particular stimuli. Being external, they can be interpersonal—they can be invoked by someone other than the person they are intended to influence, and consequently, they play a central role in administrative organization” (p. 105).

A recent review on the microfoundations of the exploration-exploitation tension (Pertusa-Ortega et al., 2021) outlines numerous antecedents which it categorizes as either individual antecedents (such as cognitive and social capabilities, risk propensity, or self-efficacy) or context-evoked antecedents perceived by organizational leaders (such as motivation and handling work stress). Our study aims to empirically test the combined effects of specific individual and context-evoked antecedents on performance in tasks that require dynamic switching between exploration and exploitation decisions.

The next two subsections of this article present an overview of key antecedents that could help explain the ability to dynamically switch between exploration and exploitation. The first subsection focuses on the individual level, and the next on the context-evoked variables—i.e., those variables that capture how an individual interacts with a context.

### Individual antecedents of exploration and exploitation

#### Personality

While people behave in reference to a specific situation, they still display considerable rank-order stability in personality traits when compared to their cohort (Bleidorn et al., 2022).

To develop our theoretical arguments, we build on the Big Five traits as outlined by McCrae and Costa (1987). This view of personality has been widely used in psychology (e.g., Soldz and Vaillant, 1999; Leutner et al., 2014) and management (e.g., Herrmann and Nadkarni, 2014; Judge and Zapata, 2015). The Big Five personality traits are defined as follows: *Conscientiousness* is the disposition “to control one’s impulses, be detail oriented and careful, and to prefer order to disorder” (Feist, 2019, p. 31). *Emotional stability* is defined as the “ability of individuals to adjust their emotional state to varied situational demands and to remain calm, levelheaded, and self-confident in stressful situations” (Herrmann and Nadkarni, 2014, p. 1,323). *Agreeableness* is the tendency “to be warm, caring, and empathetic in social relationships” (Feist, 2019, p. 31). *Openness to experience* is the disposition “to be curious and open to new experiences and ideas, and to be flexible in both behavior and thought” (Feist, 2019, p. 31). Finally, *extraversion* describes the “tendency to enjoy stimulating social activities, seek out stimulating experiences, and to be confident and leader-oriented in group settings” (Feist, 2019, p. 31). Recent evidence has shown that consciousness and openness to experience moderate the relationship between switching between exploration and exploitation and cognitive strain (Keller and Weibler, 2014). In addition, a longitudinal experiment tracking the movement of 850 individuals for a two-year period found that the Big Five personality traits partly explain exploration and exploitation in the social and spatial sphere. Extraverted individuals showed more explorative behavior and diverse routines. Openness to experience was associated with routine instability and emotional stability with routine stability (Alessandretti et al., 2018).

#### Cognitive flexibility

Cognitive flexibility—defined as the ability to appropriately adjust one’s behavior according to a changing context (Dajani and Uddin, 2015)—has been proposed as the critical cognitive ability at the organizational (Kiss et al., 2020) and individual levels (Furr, 2009; Laureiro-Martínez and Brusoni, 2018).

Management studies have investigated the cognitive antecedents of managing the switch between exploration and exploitation well. Behavioral lab studies (Laureiro-Martínez and Brusoni, 2018) and studies using fMRI (Laureiro-Martínez et al., 2015) have found that exploration and exploitation involve different cognitive processes, and leaders who recognize when to switch—and, therefore, achieve better performance—engage more brain areas and the cognitive abilities related to cognitive flexibility. In particular, the switch between exploitation and exploration relies heavily on the activation of the attention control circuitries and, therefore, higher levels of involvement of the brain’s executive functions.

In psychology, cognitive flexibility is often described as being synonymous with set- or attention-shifting. Cognitive flexibility “emerges from a complex interaction of several mechanisms” (Ionescu, 2012, p. 196). Dajani and Uddin’s (2015) conceptualization of cognitive flexibility considers multiple components, or executive functions, to provide a complete account of the mechanisms that interact and allow for cognitive flexibility to emerge. This emphasis on executive functions aligns with very recent research supporting the notion that executive functions like sustained attention (or vigilance) and working memory are the cognitive abilities that might best explain human behavior in organizations (Bergenholtz et al., 2023). We rely on Dajani and Uddin’s (2015) conceptualization of cognitive flexibility, considering salience detection, vigilance, working memory, inhibition, and switching as the central components of cognitive flexibility and, therefore, exploration-exploitation performance.

Salience detection has been described as the first step in adjusting one’s thinking to changes in the environment. Only salient stimuli attract attention, allowing us to process them further (Dajani and Uddin, 2015). The term “salient” describes “a stimulus or an aspect of a stimulus that stands out or that is set apart from others” (Uddin, 2015, p. 1). Perception and response to salient stimuli rely on the combination of sensory, visceral, autonomic, and attention systems in the brain (Uddin, 2015). Consequently, if a salient stimulus is not detected, arguably it cannot trigger a change in thinking, which would undermine adequate switching between exploration and exploitation.

Once a salient stimulus is detected, attention is allocated accordingly (Dajani and Uddin, 2015). In line with a managerial understanding of attention, vigilance and attention-switching are complementary in attentional engagement, without which effective decision-making in organizations is highly unlikely. Accordingly, vigilance stands for “attachment” to a stimulus, and executive attention for “detachment” from a stimulus (Ocasio, 2011). From a neuropsychological point of view, the term “vigilance” describes the “processes that enable sustained performance on tasks over extended periods of time” (Cohen, 2011, p. 2,440), making it a central component in most models of attention. Consequently, vigilance is a precondition for flexible thought and action in the sense that it allows one to stay focused on a task for a certain period, even if it requires switching attention between exploration-exploitation decisions.

An additional and frequently cited precondition for flexible thought and action is working memory, meaning “the short-term storage of information and its ‘online’ maintenance and manipulation” (Dajani and Uddin, 2015, p. 571). This short-term storage or updating of information enables an individual to cognitively represent multiple aspects of a complex situation, thus allowing them to select those behavioral responses that are most promising in any given situation (Kashdan and Rottenberg, 2010). Cognitive flexibility is about switching between different mental sets, and working memory provides the information processing power to uphold the information associated with different mental sets in the mind (Dajani and Uddin, 2015).

In the face of a changing environment, cognitive and behavioral responses that are no longer adequate require inhibition, making it a central component of cognitive flexibility. Hence, inhibition is a precondition for subsequent switching (Kashdan and Rottenberg, 2010; Dajani and Uddin, 2015). Inhibition is the “ability to control one’s attention, behavior, or thoughts to override competing cognitions” (Dajani and Uddin, 2015, p. 571). This ability is particularly relevant in tasks requiring frequent changes in responses and, therefore, the inhibition of previously implemented responses. That ability may be especially significant given “exploitation tends to drive out exploration” (Levinthal and March, 1993, p. 107). Therefore, we argue that inhibition is particularly important for stopping automatized exploitative behaviors and initiating explorative ones.

The final step in the process of cognitive flexibility is switching, which “involves the disengagement of an irrelevant task set and the subsequent active engagement of a relevant task set” (Miyake et al., 2000, p. 55). Switching relates to the previous antecedents in that salient internal and external stimuli attract attention, are manipulated, and indicate the cessation of a current thought or behavior, after which a shift in thought or behavior occurs (Dajani and Uddin, 2015). These explanations align with the finding that attentional switching is a fundamental mechanism for balancing exploration and exploitation (Laureiro-Martínez et al., 2015).

### Context-evoked antecedents of exploration and exploitation

An important tenet of the Carnegie approach is the understanding that decision-making is situated within an organizational context (Gavetti et al., 2007). That context is defined by specific rules and routines and conflicting goals, values, and identities; taking it into consideration when understanding a decision can limit the generalizability of scientific insights generated by studying that context but also increase their accuracy. In an overview of the past, present, and future of the Carnegie approach, Gavetti et al. (2007) stressed the importance of better understanding the impact that situational context has on organizational leaders.

In order to understand how the context affects the individual leader, we draw on Simon’s (1997) idea that environmental stimuli evoke responses if they attract attention and that we need to study the “mechanisms that allow us to allocate attention to tasks and to shift attention rapidly when a task presents itself with real-time urgency […] Motivation and emotion are the mechanisms responsible for this allocation of attention” (p. 90). We build on this idea and argue that context itself does not directly affect human behavior but does indirectly affect it through humans’ affective signals, which involve emotions and motivations. Our argument is grounded in bounded rationality, according to which context is not an objective entity as it must be perceived and defined by the individual, whereby “the steps that lead, for an actor, to his [*sic*] defining the situation in a particular way involve a complex interweaving of affective and cognitive processes” (March and Simon, 1958, 2004, p. 151). This seminal notion is in line with the state-of-the-art understanding of the “affect-as-information”-view, according to which one’s affective signals are integrated into perceptions of the environment (Schwarz and Clore, 1983; Zadra and Clore, 2011). Given that humans sense and feel contextual information before they deliberately process it, affective signals capture features from the environment that are relevant for the individual situated in it. For example, it has been found that a positive emotional state indicates that the environment is relatively harmless and that others in the social setting are allies rather than enemies (Rhoades et al., 2001).

Recent neuroscientific works on cognitive control (Krebs and Woldorff, 2017; Pessoa, 2017) and decision-making (Lerner et al., 2015) provide further support for the affect-as-information-view and add that emotional states and motivation affect cognition during decision-making through interactions with other variables, such as personality, in complex ways that are not yet fully understood (Dolan, 2002; Phelps et al., 2014; Lerner et al., 2015; Pessoa, 2017). Furthermore, and aligned with Simon’s (1967, 1997) emphasis on human adaptation, work in the cognitive sciences shows that stress is also a fundamental affective signal preparing humans to cope with challenging situations, such as difficult tasks in uncertain environments (Fink, 2016).

Thus, we treat organizational leaders’ affective signals as context-evoked antecedents that capture behaviorally relevant information from the organizational context. Specifically, we suggest *stress, emotional states*, and *task motivation* as powerful context-evoked antecedents allowing organizational leaders to direct goal-driven cognition by holding their attention on important environmental stimuli. Importantly, we consider different time horizons. Stress is an affective signal that arises as part of the context over a period that lasts beyond the task but that is nonetheless recalled in the moment of performing the decision-making task itself. Emotional states, in contrast, are felt at the moment of the task but are not directly related to it as they result from different contextual cues. Task motivation, on the other hand, captures the present affective signal driven by the immediate task environment.

#### Stress

Stress is defined as a “state of worry or mental tension caused by a difficult situation” (WHO, 2023). The purpose of this stress reaction is to prepare the human organism for either fighting a stressor or fleeing from it (Allen et al., 2014). The organizational setting rarely evokes reasons for an acute fight-or-flight response but rather results in reactions (i.e., negative feelings and thoughts) to stressful situations, such as high workload and uncertainty, that occur over a period of time, for example during a month (Sherman et al., 2012).

Under stress, cognition is impaired (Diamond, 2013). A meta-analysis conducted by Shields et al. (2016) showed that stress generally lowers switching, working memory, and cognitive inhibition, defined as selectively attending or ignoring stimuli. However, stress seems to have no negative effect on response inhibition, meaning the suppression of the dominant response. Stress also reduces cognitive flexibility by forcing attention toward highly salient stimuli related to the stressor while undermining a more top-down selection of stimulus. Stress, while decreasing cognitive control processes and increasing automatic processing, directs mental and energetical resources toward the motor control of actions (Shields et al., 2016).

The level of maturity of this stream of literature stands in contrast with the literature on the microfoundations of exploration and exploitation behavior in management, which has so far overlooked the topic of stress (see Tarba et al., 2020; Pertusa-Ortega et al., 2021). Still, there is some evidence that stress disrupts the connectivity of the frontoparietal network, temporarily undermining attention control (Liston et al., 2009), which represents the basic mechanism for exploring alternative courses of action under changing environmental circumstances (Laureiro-Martínez et al., 2015), and continuous stress at an early age can lead to an excessively exploitative decision-making approach (Humphreys et al., 2015).

#### Emotional states

Scholarly work linking emotional states to decision-making has proliferated exponentially in recent decades, increasing from practically no articles at all in 1970 to roughly 500 per year today. Nowadays, emotional states are understood as the “dominant driver” of most life-changing decisions, shaping both the content of thought and its depth (Lerner et al., 2015). We define emotional states as “complex reaction pattern[s], involving experiential, behavioral, and physiological elements, by which an individual attempts to deal with a personally significant matter or event” (American Psychological Association Dictionary of Psychology, 2023). Emotional states reflect the aggregated emotions experienced at the present moment. Importantly, while the emotions are felt in the moment, their underlying contextual stimuli may have accumulated over hours or even days (Forgas, 1995).

Overall, positive emotional states seem to favor cognitive flexibility. Several studies have found that positive emotional states decrease switching costs (e.g., Lin et al., 2013; Wang et al., 2017) while increasing working memory capacity (Levens and Phelps, 2008; Yang et al., 2013), thus favoring cognitive flexibility. Regarding inhibition, negative emotional states seem to have an effect in terms of decreasing performance (Derakshan and Eysenck, 2010). However, when considering the cognitive process of vigilance, it appears that it is not positive but rather negative emotional states that improve performance (Schwarz and Bless, 2020).

Considering the effect of emotional states on decisions about exploration and exploitation, research on the team level has shown that neither positive nor negative emotional states before taking a decision about the exploration of new routines have any effect on that decision. However, a decrease in team performance, which presumably leads to negative emotional states, before taking a decision about the adoption of a certain routine does favor exploration (Håkonsson et al., 2016). Still, on the individual level, negative emotional states seem to hinder exploration (Brusoni et al., 2020). Both our work and broader literature reviews by other authors (see Tarba et al., 2020; Pertusa-Ortega et al., 2021) indicate the need to further investigate the link between emotional states and exploration and exploitation.

#### Task motivation

Motivation, as a context-evoked antecedent, is indispensable for explaining decision-making performance (Kanfer and Ackerman, 1989). Motivation is defined as an “unobservable force that directs, energizes, and sustains behavior” (Diefendorff and Chandler, 2011, p. 66) and as “the joy of solving a task” in relation to expected rewards (Krebs and Waldorff, 2017, p. 422). Motivation affects decision-making performance by influencing the direction, intensity, and persistence of effort (Campbell, 1990). Most importantly, state-like measures of motivation, which capture immediate and transient motivation for a concrete task in a concrete setting, predict decision-making performance better than trait-like measures such as general achievement motivation (Van Iddekinge et al., 2018). Accordingly, task motivation predicts performance in a wide variety of organizations (Piccolo and Colquitt, 2006) and tasks (Freund et al., 2011).

While the association between task motivation and general decision-making performance is well established, knowledge about the role of motivation in exploration-exploitation tasks is scarce. For example, recent reviews or empirical articles related to cognitive flexibility have not considered task motivation as a factor (e.g., Ionescu, 2012; Laureiro-Martínez and Brusoni, 2018; Zmigrod et al., 2020; Howlett et al., 2021; Uddin, 2021). While the antecedent of task motivation is overlooked in much of the literature on the microfoundations of exploration and exploitation behavior (see Pertusa-Ortega et al., 2021), initial research has found that task motivation improves the dynamic switching between exploration and exploitation, presumably by increasing one’s sense of self-control and willingness to change one’s behavior in the face of a shifting environment (Mom et al., 2019). However, despite these initial findings, more evidence on the relevance of task motivation for switching between exploration and exploitation is needed, particularly in combination with cognition and personality (Tarba et al., 2020).

Table 1 provides an overview of the just-described constructs and their associations with exploration-exploitation performance.

**Table 1:** Overview of antecedents and their associations with exploration-exploitation performance.

Antecedent	Association with exploration-exploitation performance
Personality	
**Conscientiousness** is the disposition “to control one’s impulses, be detail oriented and careful, and to prefer order to disorder” (Feist, 2019, p. 31).	Personality traits, such as the Big Five (conscientiousness, emotional stability, etc.) might predict exploration and exploitation (Keller and Weibler, 2014; Alessandretti et al., 2018).
Using the term **emotional stability**, instead of neuroticism or emotional instability, we define this trait as the “ability of individuals to adjust their emotional state to varied situational demands and to remain calm, levelheaded, and self-confident in stressful situations” (Herrmann and Nadkarni, 2014, p. 1,323).	
**Agreeableness** is the tendency “to be warm, caring, and empathetic in social relationships” (Feist, 2019, p. 31).	
**Openness to experience** is the disposition “to be curious and open to new experiences and ideas, and to be flexible in both behavior and thought” (Feist, 2019, p. 31).	
**Extraversion** describes the “tendency to enjoy stimulating social activities, seek out stimulating experiences, and to be confident and leader-oriented in group settings” (Feist, 2019, p. 31).	
Components of cognitive flexibility	
**Salience detection:** Identification of “a stimulus or an aspect of a stimulus that stands out or that is set apart from others” (Uddin, 2015, p. 1).	The detection of salient stimuli allows a decision-maker to notice changes in the environment (Uddin, 2015) that might require them to switch between exploration and exploitation.
**Vigilance:** “Processes that enable sustained performance on tasks over extended periods of time” (Cohen, 2011, p. 2,440).	Vigilance allows a decision-maker to attach their attention to a stimulus (Ocasio, 2011) that might require them to switch between exploration and exploitation.
**Working memory:**“The short-term storage of information and its ‘online’ maintenance and manipulation” (Dajani and Uddin, 2015, p. 571).	Working memory allows a decision-maker to process relevant information (Dajani and Uddin, 2015) that might require them to switch between exploration and exploitation.
**Inhibition:** “Ability to control one’s attention, behavior, or thoughts to override competing cognitions” (Dajani and Uddin, 2015, p. 571).	Inhibition allows a decision-maker to stop implementing a response that is no longer adequate in a situation (Dajani and Uddin, 2015) and thus to switch between exploration and exploitation.
**Switching:**“Disengagement of an irrelevant task set and the subsequent active engagement of a relevant task set” (Miyake et al., 2000, p. 55).	Switching represents the core cognitive mechanism that allows a decision-maker to stop exploiting and start exploring or vice versa.
Context-evoked antecedents	
**Stress:**“State of worry or mental tension caused by a difficult situation” (WHO, 2023).	Stress undermines attention control (Liston et al., 2009), representing the basic mechanism for exploring alternative courses of action under changing environmental circumstances (Laureiro-Martínez et al., 2015).
**Emotional states:** “Complex reaction pattern[s], involving experiential, behavioral, and physiological elements, by which an individual attempts to deal with a personally significant matter or event” (American Psychological Association Dictionary of Psychology, 2023).	Emotional states might affect a decision-maker’s tendency to explore or exploit in a given situation (Brusoni et al., 2020).
**Task motivation:** “Unobservable force that directs, energizes, and sustains behavior” (Diefendorff and Chandler, 2011, p. 66) in a task.	Motivation affects the direction, intensity, and persistence of effort (Campbell, 1990) in tasks that might require a decision-maker to switch between exploration and exploitation.

To date, the important antecedents of exploration-exploitation have been studied separately from each other. We propose a microfoundational model that consolidates multiple antecedents to provide a more comprehensive explanation of what precedes adaptive exploration-exploitation decisions. Our model integrates the three psychological antecedents—personality, cognitive flexibility, and context-evoked antecedents—that jointly affect exploration-exploitation performance. Before turning to the integrative path model, we explain each of the three mediations in more detail.

#### The relationship between personality, cognitive flexibility, and exploration-exploitation performance

**Figure 2:**
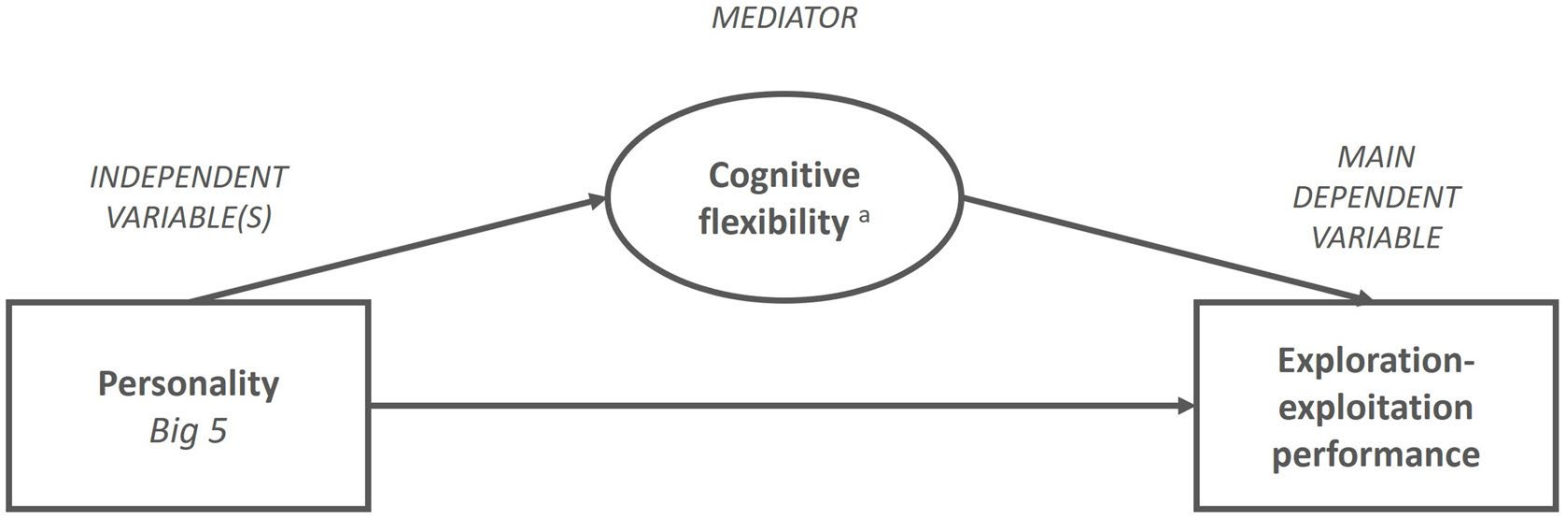
Proposed sub-model of exploration-exploitation performance in organizational leaders. P1: Cognitive flexibility mediates the effect of personality antecedents on exploration-exploitation performance. ^a^Five components of cognitive flexibility: Salience detection, vigilance, working memory, inhibition, switching.

Given the evidence about the effects of both personality and cognitive flexibility on exploration-exploitation (as described in previous sections), we theorize that both categories of antecedents are likely to affect exploration-exploitation performance. Importantly, we propose that cognitive flexibility represents the most direct influence on exploration-exploitation decisions due to the mental control efforts needed to allocate attention and process information for such tasks (Laureiro-Martínez et al., 2015). Personality antecedents, on the other hand, contribute less directly to the outcome, as they likely interact with individuals’ cognitive flexibility (Unsworth et al., 2009). In fact, personality is found to correlate with some of the components of cognitive flexibility that are critical for exploration-exploitation decisions. For example, emotional stability is associated with improved working memory, inhibition, and switching ability (Murdock et al., 2013). Openness to experience is positively associated with working memory (DeYoung et al., 2005) and switching ability (Unsworth et al., 2009; Murdock et al., 2013). Agreeableness, conscientiousness, and extraversion yield inconsistent findings (Murdock et al., 2013). In sum, some specific personality antecedents are correlated with some, but not all, components of cognitive flexibility. Given the immediate role of cognition in task performance, we propose that cognitive flexibility is likely to mediate the effect of some of the personality antecedents on exploration-exploitation performance. We do not hypothesize which specific personality antecedents or components of cognitive flexibility will have an effect but rather explore the relation summarized in this proposition (see Figure 2):

**Proposition 1 (P1):** Cognitive flexibility mediates the effect of personality antecedents on exploration-exploitation performance.

#### The relationship between recent stress, cognitive flexibility, and personality

We posit that recent stress represents a context-evoked antecedent that captures challenging organizational states that occur over a period of time. Some organizational states, such as high workload, a number of difficult tasks, and high uncertainty related to future organizational states, can evoke affective signals in the form of stress if they persist for some time. As described in the previous section, enduring stress is mostly associated with impaired cognitive flexibility. In line with these findings, we would expect to find a negative effect of recent stress on cognitive flexibility.

Importantly, however, some personality effects seem to influence the effect of recent stress. Personality, as a rather stable category of antecedents, can filter how individuals react to the influence of different contextual cues. For example, a number of studies have shown that emotional stability is associated with a lower level of experienced stress in individuals, even if potentially stressful situations endure for some time (e.g., Bibbey et al., 2013; Xin et al., 2017). Surprisingly, and in contrast to studies that only assess the direct effect of stress on decision-making performance (see previous paragraphs), it has been shown that leaders experience a lower level of stress compared to non-leaders in situations of uncertainty (Sherman et al., 2012). This finding points toward a view that leaders possess personality traits, such as emotional stability, that make them less reactive to stress in times that evoke considerable stress and negative emotions in others. In contexts that involve high uncertainty and impose cognitive load, such as the exploration-exploitation dilemma, personality antecedents such as emotional stability are, therefore, likely to filter the kind or scope of potentially detrimental affective signals evoked by the context.

**Figure 3:**
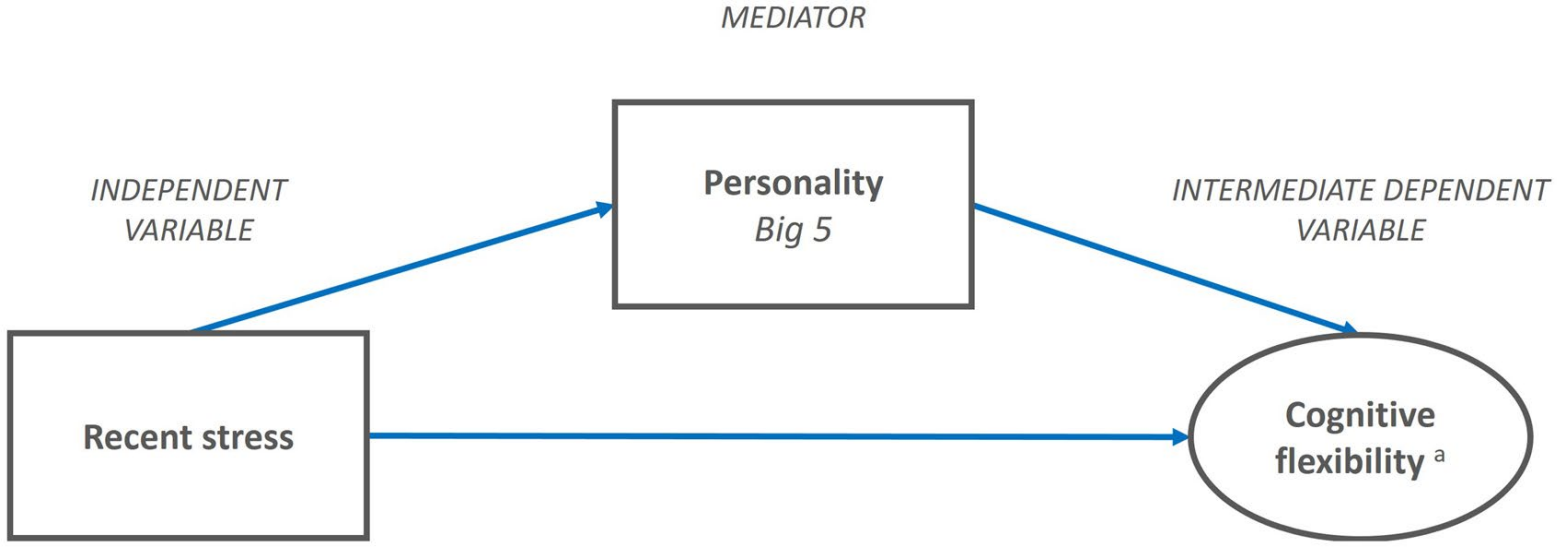
Proposed sub-model of exploration-exploitation performance in organizational leaders. P2: Personality antecedents mediate the effect of recent stress on cognitive flexibility. ^a^Five components of cognitive flexibility: Salience detection, vigilance, working memory, inhibition, switching.

Thus, personality antecedents such as emotional stability are likely to influence the level of activation of negative and sustained stress by filtering the interpretation of contextual cues—an effect that is captured by the perceived level of (sustained) stress. As with Proposition 1, we do not hypothesize ex-ante which specific Big Five personality antecedents act as mediators but will empirically test this. Taken together, we postulate an indirect relationship summarized in this proposition (see Figure 3):

**Proposition 2 (P2):** Personality antecedents mediate the effect of recent stress on cognitive flexibility.

#### The relationship between present context antecedents, cognitive flexibility, and exploration-exploitation performance

Neuroscientific studies have found evidence that affective signals do not necessarily lead to flawed cognition and biased decisions but favor awareness of important contextual cues—in contrast to what has been posited in traditional philosophy and economic theories of rationality (Dolan, 2002). Lerner et al. (2015) proposed that context antecedents, such as emotions, that have motivational quality can indirectly influence decision-making outcomes by influencing, for example, the type of cognitive processes used (e.g., analytic vs. heuristic thinking). Pessoa (2017), using motivation as an example of an affective signal, summarized three options relevant for tasks that require cognitive flexibility: (1) affective signals and cognition could co-evolve in parallel during an event but still contribute separately to the outcome (model “parallel”); (2) cognition could mediate affective signals so that it changes the effect of the affective signals on the outcome (model “mediation”); and (3) affective signals and cognition are truly integrative, in that they are not separable (model “integration”).

**Figure 4:**
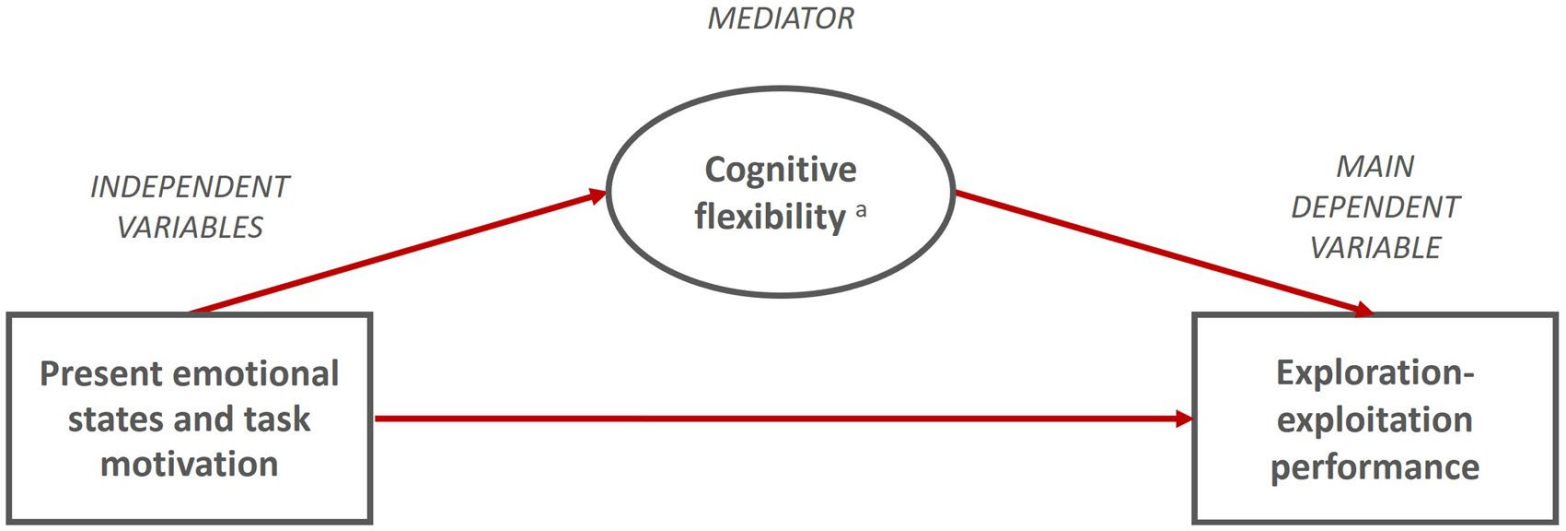
Proposed sub-model of exploration-exploitation performance in organizational leaders. P3a-b: Cognitive flexibility mediates the effect of present emotional states (3a) and task motivation (3b) on exploration-exploitation performance. ^a^Five components of cognitive flexibility: Salience detection, vigilance, working memory, inhibition, switching.

Given that cognitive flexibility is expected to affect exploration-exploitation most directly, we propose a mediation effect between present context antecedents and cognitive flexibility. To give an illustrative example: Positive emotional states and task motivation typically have a positive effect on task outcomes (Van Iddekinge et al., 2018). In addition, as outlined in our model, it has been argued that affective signals (i.e., emotional states and task motivation) also influence cognitive flexibility, which, in turn, might change and mediate the effect of positive affective signals on task performance by engaging the same set of cognitive functions (see Pessoa, 2017). We expect positive emotional states and task motivation to have positive indirect effects on exploration-exploitation and negative emotional states to have negative indirect effects. In both cases, we assume the same kind of indirect relationship. In line with our argumentation, we make the following propositions (see Figure 4):

**Proposition 3a (P3a):** Cognitive flexibility mediates the effect of the present emotional state on exploration-exploitation performance.

**Proposition 3b (P3b):** Cognitive flexibility mediates the effect of present task motivation on exploration-exploitation performance.

**Figure 5:**
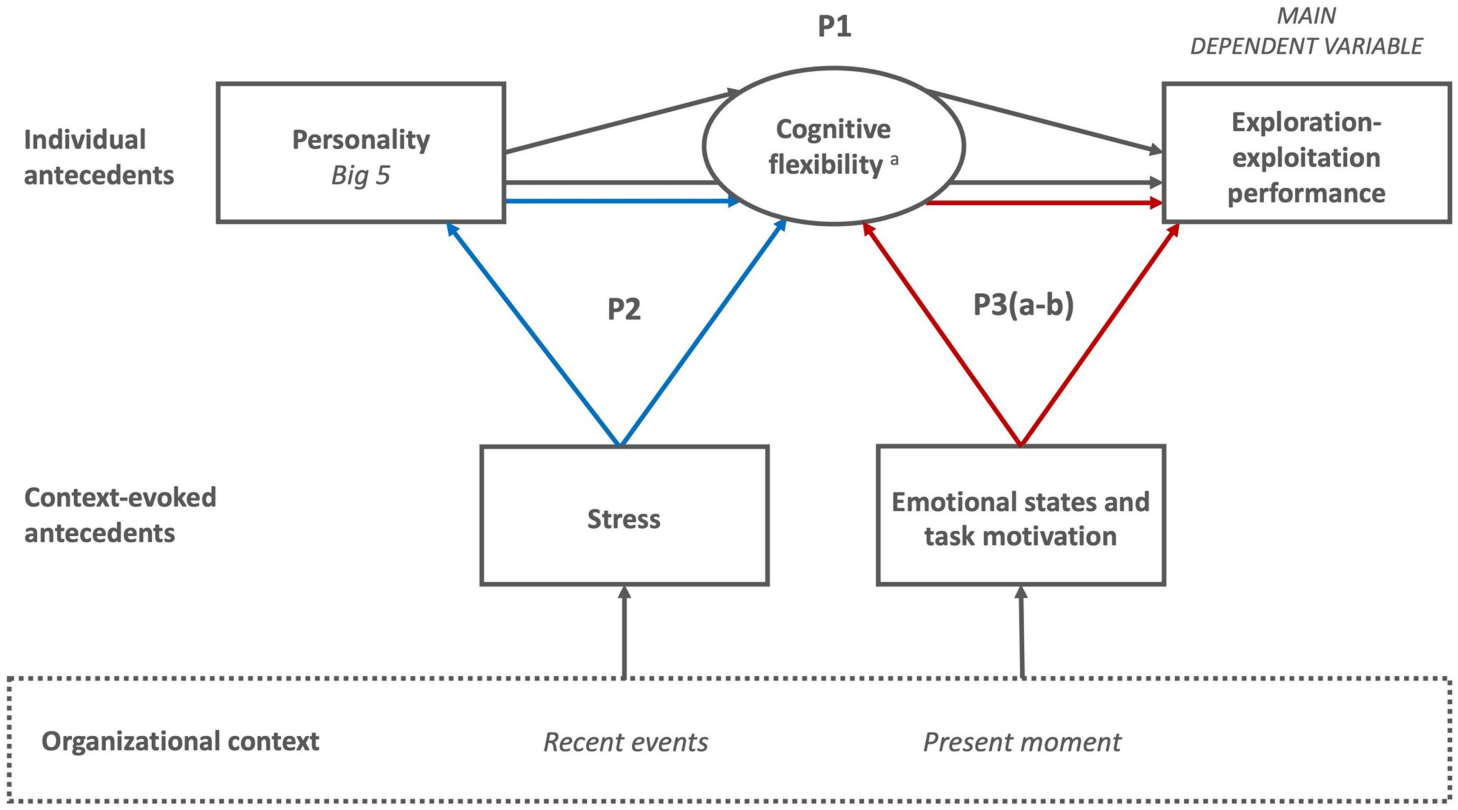
Proposed integrative path-model of exploration-exploitation performance including all three mediations. ^a^Five components of cognitive flexibility: Salience detection, vigilance, working memory, inhibition, switching.

Based on our propositions, we present a model (see Figure 5) of exploration-exploitation performance that includes three connected mediations with different types of antecedents. The model takes into account two levels of antecedents (i.e., individual and context-evoked antecedents) and different time horizons (i.e., recent and present). In order to include all three mediations into one model, we slightly changed the arrangement of the three mediation effects.

## Materials and methods

### Sample and data collection

We examined the propositions described above with a lab-in-the-field approach conducted in officer schools of the Swiss Armed Forces. This means that we employed laboratory equipment to collect our data in the study participants’ working environment, favoring the external validity of our findings (Gneezy and Imas, 2017). Data collection took place in the facilities of each of the participating officer schools between August 2021 and September 2022. The participants used their personal laptops provided by the officer school to access the experimental online platform Gorilla.sc. This approach of collecting data in the cadets’ own facilities, where they were surrounded by their colleagues and submitted their responses via the laptops they used in daily organizational life, allowed us to better capture context-evoked variables that unfold in a situated manner and over different periods of time. This favors the external validity of our findings (Gneezy and Imas, 2017). Participants answered our questions, conducted tests, and made decisions in sessions of around two hours each. To reflect our theoretical model subdividing context-evoked antecedents into recent past (i.e., stress occurring over a month) and present (i.e., present emotional states and task motivation) variables, participants first answered the questions regarding their emotional states and perceived stress around one hour before taking the exploration-exploitation decisions, followed by questions related to their task motivation around 10 min before that. The individual antecedents were measured between (components of cognitive flexibility) stress and task motivation and after (personality) exploration-exploitation performance. The data on the two control variables, gender and age, were collected and transmitted by the officer schools around one month before our data collection.

The sample consisted of 282 officer cadets undergoing a 15-week officer training program. Officer schools prepare cadets to act as leaders of groups of 30 soldiers and five sergeants in often uncertain and hostile environments. The training includes the acquisition of competencies such as tactical leadership, team management, medical first aid, and survival (Eidgenössisches Department für Verteidigung, Bevölkerungsschutz und Sport, 2023). About 93% of our study participants were male, and their average age was 24. All had undergone basic military training in the Swiss Armed Forces as well as an extensive selection process before becoming officer cadets. They had also all either finished vocational training or achieved the general qualifications for university entrance before starting basic military training. The participants’ superior officers (their “commanders”) requested that participants join our study information session. In this session, we incentivized participants to perform at their best by emphasizing that the results would help to improve officer selection in the Swiss Armed Forces and that the usefulness of the data collection depended on their effort in the behavioral tasks and their willingness to provide self-reports that reflected their genuinely honest self-assessment. Confidentiality was assured and participation was voluntary. Over 95% of all addressed officer cadets chose to take part in the study. We excluded 20 participants from the data set, reducing the sample from 302 to 282 (7% of total participants), as their results indicated low motivation for taking part in the data collection. Apart from these twenty participants, behavior during and immediately after data collection (e.g., asking questions afterward and staying longer than planned to finish data collection), as well as the consistency of the results across similar variables, indicates that the sample was, on average, highly motivated to provide data that reflected their “true” level of ability and self-assessment.

### Measures

#### Personality

We used the German version of the *Big Five Inventory 2* (BFI-2; Soto and John, 2017) to measure personality, meaning the Big Five personality traits. Exemplary items were as follows: “I am someone who is…” “…dependable, steady” (*conscientiousness*), “…relaxed, handles stress well” (*emotional stability*), “…compassionate, has a soft heart” (*agreeableness*), “…curious about many different things” (*openness to experience*), or “…outgoing, sociable” (*extraversion*). The items were scored on a five-point Likert scale. Cronbach’s Alpha for the different subscales were 0.85 for *conscientiousness*, 0.85 for *emotional stability*, 0.80 for *agreeableness*, 0.80 for *openness to experience*, and 0.84 for *extraversion*.

#### Cognitive flexibility

We relied on Dajani and Uddin’s (2015) conceptualization of cognitive flexibility and measured five executive functions as the central components of cognitive flexibility.

##### Salience detection

We measured salience detection through the *visual search task* (Stoet, 2011; Mallik et al., 2020). This task included 50 trials in which participants were asked to respond if the target stimulus, an upright orange letter T, was shown on the screen and not to respond if it was not. After four seconds without a response, the trial was terminated as “no response.” Distracting stimuli were blue Ts presented in various orientations and orange Ts in opposing orientations. There were conditions with five, 10, 15, or 20 distractor stimuli, and half of the trials did not include the target stimulus. Erroneous responses were indicated with a red cross shown for two seconds. The outcome variables of the task were *accuracy*, *average reaction time*, *average reaction time with 5, 10, 15, or 20 distractors on the screen*, and *slope*, calculated through a linear regression with the set size of distractors as the independent variable. We used the task variable *slope* for our statistical analyses.

##### Vigilance

We measured vigilance through the *Mackworth clock test* (Mackworth, 1948; Vujic, 2017), a typical task for assessing vigilance. For around five minutes, participants watched a clock hand ticking around a dial. When the hand jumped forward two increments instead of the usual one, participants had to immediately press the space bar; otherwise, they were instructed to do nothing. If they were correct, a green light was shown; when incorrect, a red light was shown. Each of the five circuits dial consisted of 60 ticks, with 15 two-step jumps to identify and report. The outcome variables of the task were *number of correct answers*, *false alarms*, *actual misses*, *all misses*, and *reaction time for correct answers*. We used the task variable *number of correct answers* for our statistical analyses.

##### Working memory

We measured working memory through the *n-back task* (Kirchner, 1958; Laureiro-Martínez, 2014). In this task, participants need to indicate by pressing two different keys whether they have seen a given letter two positions earlier in a sequence. The letter could be written in lower or upper case. The n-back task included 35 trials. The outcome variables of the task were *number of correct answers*, *mistakes*, *misses*, *reaction time for mistakes*, and *reaction time for correct answers*. We used the task variable *number of correct answers* for our statistical analyses.

##### Inhibition

We measured inhibition through the *Stroop task* (Miyake et al., 2000; Moore and Malinowski, 2009). In this task, participants were shown the names of colors printed in a congruent color (e.g., the word “blue” in blue text) and an incongruent color (e.g., the word “blue” written in red), and they had to resist the automatized response to indicate the meaning of the word rather than the color of the text. The control condition included a string of five asterisks in place of a word. After 24 practice trials, the main task consisted of 72 trials with a five-asterisk string printed in one of four colors (red, green, blue, or purple), 60 trials in the incongruent condition, and 12 trials in the congruent condition. The outcome variables of the task were *number of errors*, *anticipations*, *reaction time incongruent condition*, *reaction time asterisks*, and *reaction time difference*. *Reaction time difference* was calculated through the difference between the incongruent and asterisks conditions. We used the task variable *reaction time difference* for our statistical analyses.

##### Switching

We measured switching through the *number-letter task*. In this task, a number-letter pair (e.g., “4 K”) is shown in one of four quadrants (Miyake et al., 2000). The participants were asked to indicate whether the number was odd or even when the number-letter pair was shown in one of the upper two quadrants. When the number-letter pair was shown in one of the lower two quadrants, participants had to indicate whether the letter was a consonant or a vowel. The first two blocks included 32 trials each. In a subsequent third block of 128 trials, the number-letter pair rotated in a clockwise manner around all four quadrants. Hence, the trials in the first two blocks did not require participants to switch between tasks (“single trials”), but in half of the trials in the third block, they had to conduct these two different types of categorization operations quickly and correctly (“mixed trials”). The outcome variables of the task were *number of correct answers in single trials*, *total number of correct answers in mixed trials*, *number of correct answers in mixed trials with a switch*, and *number of correct answers in mixed trials without a switch*. All these variables were also calculated based on reaction time. We used the task variable *number of correct answers in mixed trials with a switch* for our statistical analyses.

#### Context-evoked antecedents

##### Stress

We used the German version (Schneider et al., 2020) of the *perceived stress scales* to measure context-evoked stress. The 10-item scale includes the subscales of helplessness and self-efficacy. They capture perceived stress over the past month, i.e., the recent past. Exemplar items were “In the last month, how often have you felt that things were going your way?” (self-efficacy) and “In the last month, how often have you felt that you were unable to control the important things in your life? (helplessness). The items were scored on a five-point Likert scale. Cronbach’s Alpha for this study was 0.83.

##### Emotional states

We measured context-evoked emotional states through the German version of the *positive and negative affect schedule* (PANAS). The 20 items are subdivided into 10 positive and 10 negative affect items, which are scored on a five-point Likert scale (Breyer and Bluemke, 2016). They capture the emotional states of the participants “in the moment,” meaning the present. Exemplar items were “active,” “interested,” “excited” (positive affect), “distressed,” “guilty,” or “scared” (negative affect). Cronbach’s Alpha for *positive affect* was 0.86 and for *negative affect* it was 0.72.

##### Task motivation

We measured task motivation through the *current achievement motivation questionnaire*, including 18 items representing the four factors of anxiety, challenge, interest, and probability of success. They capture the motivation perceived about a task right before performing it. Exemplar items were “I feel under pressure to do this task well” (anxiety), “I am really going to try as hard as I can on this task” (challenge), “I would work on this task even in my free time” (interest), and “I think I am up to the difficulty of this task” (probability of success). The items were scored on a seven-point Likert scale (Freund et al., 2011). Cronbach’s Alpha for this study was 0.75.

#### Exploration-exploitation performance

We used the *four-armed bandit task* to measure exploration-exploitation performance, which is a standard task in strategic management literature used to measure dynamic switching between exploration and exploitation (Laureiro-Martínez et al., 2015). In the four-armed bandit task, the subject sees four differently colored slot machines, with each slot representing unknown payoff probabilities. The participant’s objective is to achieve the highest final payout possible. During the task, the slots’ payoff probabilities continually change, and the subjects must choose whether they want to continue playing on the current slot or switch to another one. The choices made by subjects imply trade-offs between gleaning more information about the payout of each slot (exploration) and using available information to collect a payout (exploitation)—known as a “sequential choice problem” (Posen and Levinthal, 2012). The task consists of 150 trials subdivided into two blocks of 75 trials each. In line with the literature (see above), *total payout* is the main outcome variable of this task and represents exploration-exploitation performance.

The rationale for choosing such a task in our lab-in-the-field study within the Swiss Armed Forces was its comparability to their central activity: preparing for the unlikely event of war. War simulations such as war gaming and urban warfare reconstructions are considered a central training activity in military leadership education (Hirst, 2022). Such simulations allow leaders to generate and test strategic decisions in the form of collective search processes and have been described as not only the best but also the *only* form of training (Augier et al., 2018). According to Robert Work, a former U.S. Deputy Secretary of Defense, war simulations “provide structured, measured, rigorous […] environments to help us explore what works (winning) and what doesn’t (losing) across all dimensions of warfighting” (Hirst, 2022, p. 5).

“Memoir 44” is an exemplary board game for war simulation that some of the officer schools participating in our study used to train tactical decision-making in their cadets. It thematizes the battles of World War II, is played on a hexagon-gridded board (the “battlefield”), and relies on the successful use of military principles and procedures (“options”) to defeat the opponent (Borg, 2004). Memoir 44 captures exploration-exploitation performance as it requires its players to execute standardized tactical options and to stick to them if they serve the given military objective (i.e., exploitation) and to change or creatively combine tactical options when they do not (i.e., exploration). In addition to Memoir 44, we observed how some officer schools simulate urban warfare exercises. The reconstruction and usage of a realistic environment and equipment intensify the dynamic interaction between the different actors and evoke affective signals that are included in decision-making. Especially under such realistic situations, decision-makers need to dynamically shift between the exploitation of given options and the exploration of new ones.

The four-armed bandit task is well suited to capturing the essence of the underlying tension between the exploration and exploitation behaviors that occur in war simulations. Furthermore, in both tasks, learning reduces uncertainty and increases the decision-maker’s probability of success. While the two task paradigms differ in some respects—e.g., the war simulations feature more behavioral options and require more previous knowledge than the four-armed bandit task—they both allow for observation of exploration-exploitation performance. As such, the four-armed bandit task captures our dependent variable well. Table 2 outlines this comparison in more detail.

**Table 2:** Comparison of war simulation exercises and this study’s lab-in-the-field task.

	War simulations	Four-armed bandit task
**Objective**	Gain urban terrain (i.e., take terrain from opponent) by choosing the most effective attack options.	Maximize income by choosing the options with the highest possible payoffs.
**Available options**	Choose the most effective tactical option (e.g., line vs. column or combination of both) and potentially use other creative moves within these basic options.	Choose the most effective option among four given options (four slots).
**Uncertainty**	High in the beginning; learning reduces uncertainty to some degree as some patterns and facts are recognizable (opponent’s tactics and reactions, not everything in the terrain is visible at the beginning but becomes clearer).	High in the beginning, learning reduces uncertainty to some degree as some patterns and facts are recognizable (how high the payoffs can be, increasing/decreasing patterns).
**Description of task structure**	Well-structured regarding the goal, the presence of basic choice options (e.g., tactical formation options: line vs. column or combination), and basic warfare principles, but ill-structured regarding other aspects (e.g., leaders must not only make decisions for themselves but also for others; information regarding opponent is incomplete; the environment is changing; the setting allows leaders and opposing leaders to come up with unexpected moves, such as creative attack ideas within the basic options although they do not occur frequently).	Well-structured regarding the goal and presence of clear options to choose from, but ill-structured regarding the dynamic and not always predictable changes in the environment.
**Task characteristics**	Main task characteristics evoking the allocation of attention include task instruction, urban terrain, weather conditions, superiors, own troops, enemy, condition of required items (food, radio, vehicle, weapons, etc.) and binding regulations.	Main task characteristics evoking the allocation of attention include task instruction, used computer, visual representation of four-armed bandits in different colors and varying payoffs provided by different bandits.
**Behaviors**	Explore or exploit the given options.	Explore or exploit the given options.
**Exploitation**	Exploitation is favored when the current option (considering available human, material, and time resources) is believed to bring the troops closer to their military objective.	Exploitation is favored when the current option is believed to offer the highest payoff.
**Exploratio**n	Exploration is favored when the current option is not believed to bring the troops closer to their military objective and, instead, an alternative option is believed to be better.	Exploration is favored when an alternative option is believed to offer a higher payoff.
**Required previous knowledge**	Some, leaders need awareness of the basic principles of warfare and tactical options.	None, all participants were exposed to the same payoff instantiation for the first time.
**End state**	Typically ends when one party has achieved its military objective.	Ends after a predetermined number of trials (150).

For the control variables of *gender* and *age*, we relied on information already collected by the organization at the time the individuals joined it.

## Results

### Descriptive statistics

In Table 3 we provide the descriptives of our study. To handle extreme outliers’ values (more than 3 standard deviations from the average), we used a method called Winsorizing (Field, 2013). Our data didn’t follow a normal distribution, so we used Spearman correlations. We did not only consider significant *p*-values in the analysis of our data but also the strength of correlations. If a correlation was not significant at the 0.05 level but seemed relevant, we report the exact value of *p*. Moreover, we could not include *negative* emotional states due to a lack of variance but kept *positive* emotional states in our analyses.

**Table 3:** Descriptives and Spearman correlations among study variables (*N* = 282).

Variables		*M*	SD	1	2	3	4	5	6	7	8	9	10	11
1.	Exploration-exploitation performance	8,998	637											
2.	Gender^a^	0.07	0.26	−0.16^**^										
3.	Age	23.7	3.3	0.14^*^	0.00									
4.	Conscientiousness	3.82	0.51	−0.03	0.07	0.02								
5.	Emotional stability	3.73	0.53	0.16^**^	−0.14^*^	0.11	0.46^**^							
6.	Agreeableness	3.79	0.48	0.08	−0.03	0.00	0.31^**^	0.37^**^						
7.	Openness to experience	3.54	0.56	0.05	0.12^*^	0.06	0.19^**^	0.16^**^	0.21^**^					
8.	Extraversion	3.62	0.52	−0.02	0.05	−0.11	0.34^**^	0.31^**^	0.16^**^	0.31^**^				
9.	Stress	2.47	0.54	−0.13^*^	0.07	−0.17^**^	−0.31^**^	−0.55^**^	−0.09	−0.17^**^	−0.20^**^			
10.	Emotional states	3.20	0.60	−0.04	0.00	−0.03	0.21^**^	0.26^**^	0.16^**^	0.19^**^	0.21^**^	−0.26^**^		
11.	Task motivation	4.03	0.73	0.11	0.05	0.11	−0.03	0.06	0.08	0.26^**^	−0.04	−0.08	0.18^**^	
12.	Salience detection (msec.)^b,c^	−25	11.90	0.02	0.02	−0.00	0.07	0.01	0.05	0.01	−0.01	0.02	0.05	0.04
13.	Vigilance^b^	11.10	2.84	0.14^*^	−0.08	0.15^*^	0.13^*^	0.16^**^	0.08	0.25^**^	−0.01	−0.10	0.16^**^	0.19^**^
14.	Working memory^b^	27.40	6.96	0.25^**^	−0.08	0.04	0.02	0.04	0.02	0.06	−0.04	−0.06	0.09	0.19^**^
15.	Inhibition (msec.)^b,c^	−123.85	61.57	−0.04	0.01	−0.08	−0.07	−0.02	0.02	−0.03	−0.06	0.14^**^	−0.00	0.05
16.	Switching *^b^*	59.46	4.82	0.16^**^	0.08	0.18^**^	0.16^**^	0.14^*^	0.09	0.11	0.10	−0.10	−0.00	0.13^*^
17.	Cognitive flexibility^d^	-	-	0.43^***^	0.01	0.27^**^	0.24^*^	0.28^**^	0.06	0.35^**^	0.03	−0.19	0.22	0.45^***^
Variables				12	13	14	15	16						
13.	Vigilance			0.08										
14.	Working memory			0.11	0.19^**^	-								
15.	Inhibition (msec.)			0.12^*^	0.05	0.03								
16.	Switching			−0.00	0.18^**^	0.13^*^	−0.09							
17.	Cognitive flexibility *^d^*			0.08	-	-	−0.01	-						

^a^Gender: male = 0, female = 1. Sample includes 20 female participants. ^b^Components of cognitive flexibility. ^c^Msec., milliseconds, where a low number equals high performance. ^d^For latent factors of cognitive flexibility including vigilance, working memory, and switching see factor loadings above. ^*^p < 0.05; ^**^p < 0.01; ^***^p < 0.001.

Regarding the control variable of gender and age, our findings show a negative correlation between being female and exploration-exploitation performance. A partial explanation for this connection might lie in the negative relationship between emotional stability and being female. Still, it is important to note that our sample only includes 20 female participants, and the difference in exploration-exploitation performance is relatively small (female *M* = 8,702, male *M* = 9,021). As a result, we will report all model fits and mediation effects (see Tables 4, 5) without gender. However, we did include whether the model fits with gender as a robustness check.

**Table 4:** Standardized effects of one-mediator path model (Proposition 1).

Model pathway	Direct effect	Indirect effect	*p*	95% CI
Emotional stability ➔ Cognitive flexibility ➔ Exploration-exploitation performance	0.045 (*p* = 0.495)	0.244 × 0.472 = 0.115	0.003	52.218, 368.125

**Table 5:** Standardized effects of two-mediator path model (Propositions 2 and 3b).

Model pathways	Direct effect	Indirect effect	*p*	95% CI
Stress ➔ Emotional stability ➔ Cognitive flexibility	0.016 (*p* = 0.862)	−0.584 × 0.257 = −0.150	0.004	−1.489, −0.212
Task motivation ➔ Cognitive flexibility ➔ Exploration-exploitation performance	−0.100 (*p* = 0.234)	0.444 × 0.544 = 0.242	0.000	77.881, 421.539

Three out of the five components of cognitive flexibility (vigilance, working memory, and switching) positively correlate with each other.

Exploration-exploitation performance correlates positively with emotional stability, task motivation (*p* = 0.057), and cognitive flexibility and negatively with stress. However, it does not correlate with emotional states. Cognitive flexibility, in turn, correlates positively with age, emotional stability, emotional states (*p* = 0.136), and task motivation and negatively with stress (*p* = 0.058).

We will not test Proposition 3a as emotional states do not correlate with exploration-exploitation performance. We consider stress and task motivation as relevant for our sample, even if their correlations with exploration-exploitation and cognitive flexibility, respectively, are slightly below the 0.5 value of *p* threshold. There is very strong evidence for the notion that stress has a negative effect on human cognition and, overall, cognitive flexibility. We outlined a fraction of this evidence. Similarly, task motivation has been shown to influence decision-making across various contexts and samples.

We found further associations between our study variables. For instance, consciousness and openness to experience are correlated with cognitive flexibility and stress. Likewise, openness to experience is correlated with task motivation. While these findings provide insights into how personality and context-evoked antecedents are related and impact cognitive flexibility, they fall outside the theoretical scope of this article. Therefore, we will not consider them further.

### Path models

We tested our propositions through one-mediator (Proposition 1) and two-mediator (Propositions 2 and 3b) path models using structural equation modeling with Amos SPSS 28 (see Collier, 2020; Arbuckle, 2021). We conducted a maximum likelihood estimation and report standardized regression coefficients to quantify the strength of the mediations within the two models as well as bootstrapping analysis with 5,000 random samples to test the indirect effects (Hayes, 2013).

The correlations between vigilance, working memory, and switching indicate the possibility of building a factor variable for cognitive flexibility through structural equation modeling (Collier, 2020; Arbuckle, 2021). According to recommendations in the field of strategic management (Hair et al., 2012) and psychology (Gong et al., 2020; Hu et al., 2021), factor loadings should lie above 0.50. This applied to vigilance and switching. However, a strong theoretical rationale and overall model fit can justify the inclusion of factor loadings between 0.30 and 0.40 (Brown, 2015; Smedslund et al., 2022). Theory and empirics (Miyake et al., 2000; Ionescu, 2012; Diamond, 2013) clearly support the notion that working memory is a component of cognitive flexibility, and our model fits were very good to excellent, including working memory. Vigilance showed a factor loading of 0.54 in the one-mediation analysis of Proposition 1, and 0.50 in the two-mediation analysis combining Propositions 2 and 3. Switching showed a factor loading of 0.52 (one-mediation) and 0.54 (two-mediation). And working memory showed a factor loading of 0.36 (one-mediation) and 0.36 (two-mediation).

We assessed the overall fit of the models based on the following indices (Hu and Bentler, 1999; Zyphur et al., 2023): chi-square statistic (χ^2^), df, the standardized root mean square residual (SRMR) ≤ 0.08, the root mean square error of approximation (RMSEA) ≤ 0.06, the comparative fit index (CFI) ≥ 0.95, and the Tucker-Lewis index (TLI) ≥ 0.95.

First, we tested a path model in which age, emotional stability, stress, task motivation, and cognitive flexibility are directly associated with exploration-exploitation performance. This model provided an unsatisfactory fit to the data: χ^2^(10, *N* = 282) = 148.013, *p* = 0.000; SRMR = 0.132; RMSEA = 0.222, CFI = 0.348, TLI = −0.369. Note that no model referencing our propositions and including direct links to exploration-exploitation performance shows a satisfactory fit.

**Figure 6:**
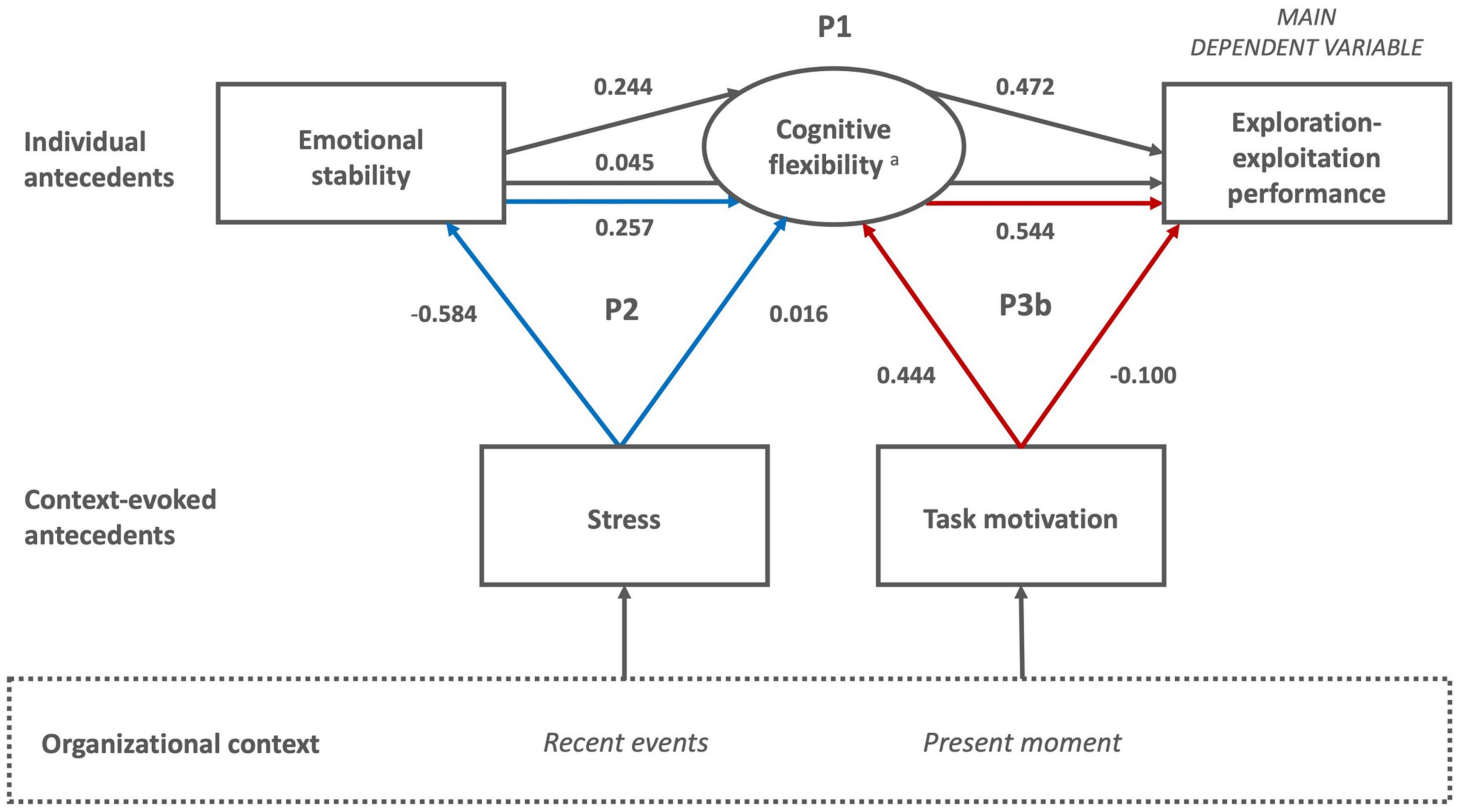
Integrative path-model of exploration-exploitation performance including results for the Propositions 1, 2, and 3b. ^a^Cognitive flexibility is based on vigilance, working memory and switching. For simplicity, the control variable of age is not included into the figure. All shown parameters are standardized. All indirect effects are significant, and the direct effects insignificant representing full mediations.

Second, we ran a path model to test Proposition 1, including the variables of age, emotional stability, cognitive flexibility, and exploration-exploitation performance. The fit of this model was very good: χ^2^(6, *N* = 282) = 7.187, *p* = 0.304; SRMR = 0.030; RMSEA = 0.027, CFI = 0.986, TLI = 0.966. The mediation effect showed that cognitive flexibility significantly mediates the relationship between emotional stability and exploration-exploitation performance (β = 0.115, *p* = 0.003; mediation path a and b) and there remains an insignificant direct effect of emotional stability on exploration-exploitation performance (β = 0.045, *p* = 0.495; mediation path c’). The bootstrap analysis showed that there is no zero in the 95% CI for the estimates, confirming a mediation effect (Preacher and Hayes, 2008; Proposition 1 accepted). Note that all effects reported in this results section are standardized. The mediation analysis of Proposition 1 is presented in Table 4 and Figure 6.

Third, we ran the two-mediation path model to test Propositions 2 and 3b, including the variables of age, stress, emotional stability, cognitive flexibility, task motivation, and exploration-exploitation performance. This model showed an excellent fit: χ^2^(14, *N* = 282) = 10.742, *p* = 0.767; SRMR = 0.027; RMSEA = 0.000, CFI = 1.000, TLI = 1.028. Considering Proposition 2, the results show that emotional stability significantly mediates the relationship between stress and cognitive flexibility (β = −0.150, *p* = 0.004; mediation path a and b) and that there is an insignificant (remaining) direct effect of stress on cognitive flexibility (β = 0.016, *p* = 0.862; mediation path c’). The bootstrap analysis additionally confirmed a mediation effect (Preacher and Hayes, 2008). Thus, although the initial correlation between stress and cognitive flexibility did not meet the 0.5 value of *p*, the path model resulted in a full mediation (Proposition 2 accepted). Considering Proposition 3b, the results showed that cognitive flexibility mediates the relationship between task motivation and exploration-exploitation performance (β = 0.242, *p* = 0.000; mediation path a and b) and that there is an insignificant (remaining) direct effect of task motivation on exploration-exploitation performance (β = −0.100, *p* = 0.234; mediation path c’). The bootstrap analysis confirmed the mediation effect (Preacher and Hayes, 2008). Again, although the initial correlation between task motivation and exploration-exploitation did not meet the 0.5 value of *p*, the path model resulted in a full mediation (Proposition 3b accepted). The two-mediation path model for Propositions 2 and 3b is presented in Table 5 and Figure 6.

Based on guidelines (Gignac and Szodorai, 2016) for assessing beta weights on the individual level of analysis, the identified mediation effects are small (Proposition 1) to moderate (Propositions 2 and 3b). Hence, while being meaningful, the outlined mediation effects can only explain a small to moderate amount of variance in our model.

### Robustness checks

To test the robustness of our findings, we conducted four additional analyses, all of which supported our results.

First, an alternative explanation to Proposition 1 is that emotional stability mediates the relationship between cognitive flexibility and exploration-exploitation performance. This mediation effect is clearly insignificant (*p* = 0.356).

Second, an alternative explanation to Proposition 2 is that stress affects cognitive flexibility under the condition of low emotional stability. We tested this assumption through a moderation analysis, which was clearly insignificant (*p* = 0.924). This means that emotional stability is a mediator as outlined in Proposition 2, not a moderator.

Third, the direct correlation between stress and exploration-exploitation performance might challenge the notion that stress affects such performance through cognitive flexibility as implied in Proposition 2. Hence, we included a direct link between stress and exploration-exploitation performance in the two-mediation analysis and found that this correlation disappears (*r* = −0.009, *p* = 0.896). This means that the direct correlation between stress and exploration-exploitation performance is based on the effect that stress has on cognitive flexibility, which then affects exploration-exploitation performance.

Fourth, we tested alternative explanations for the role of emotional states in our model. We excluded emotional states from Proposition 3 because they are not related to exploration-exploitation performance, contradicting a mediation effect. However, given the insignificant but noteworthy correlation between emotional states and cognitive flexibility (*r* = 0.22), we tested whether emotional stability mediates the relationship between emotional states and cognitive flexibility. This analysis led to an unsatisfactory model fit: χ^2^(14, *N* = 282) = 32.919, *p* = 0.003; SRMR = 0.054; RMSEA = 0.069, CFI = 0.877, TLI = 0.754. This finding provides support for the notion that emotional states play a subordinated role in the explanation of exploration-exploitation performance.

Fifth, we included gender in the one-mediation (Proposition 1) and two-mediation (Propositions 2 and 3b) analysis. This led to a just sufficient fit in the one-mediation [χ^2^(3, *N* = 282) = 6.996, *p* = 0.072; SRMR = 0.029; RMSEA = 0.069, CFI = 0.948, TLI = 0.741] and a good fit in the two-mediation [χ^2^(8, *N* = 282) = 8.366, *p* = 0.399; SRMR = 0.027; RMSEA = 0.013, CFI = 0.998, TLI = 0.994]. This means that our results also hold if we include the control variable of gender.

Our findings support our theoretical model, leading to the following conclusion: emotionally stable leaders perform better on exploration-exploitation decisions than their less emotionally stable counterparts. This advantage is based on the favorable effect that emotional stability has on cognitive flexibility (Proposition 1). Context-evoked stress occurring over a recent period of time has a negative effect on cognitive flexibility, as one might expect, but importantly, emotional stability decreases this detrimental effect (Proposition 2). Likewise, a leader’s present task motivation positively affects exploration-exploitation performance, but this effect is fully mediated by cognitive flexibility (Proposition 3b). Interestingly, our results indicate that present emotional states are not related to exploration-exploitation performance. This finding suggests that present and positive affective signals only play a role if they are directly relevant to the task.

## Discussion

Congruent with the Carnegie approach, our study examined a central issue in everyday organizational life: the need to dynamically switch between exploratory and exploitative decisions to adapt to the environment. Organizations must rely on leaders who manage exploration and exploitation in an adaptive way by making decisions appropriate to a given context at any moment in time.

We proposed a model that situates exploration-exploitation decisions in context. Drawing on March and Simon’s (1958, 2004) view of a complex interaction between affective and cognitive processes, our integrative model included individual antecedents (i.e., personality and cognitive flexibility) as well as context-evoked antecedents with different time horizons that capture how leaders rely on affective signals to interpret the organizational context (i.e., stress, emotional states, and task motivation). We relied on a lab-in-the-field study to test our model with a sample of leaders taking part in training and practicing leadership skills within the Swiss Armed Forces. First, we identified cognitive flexibility as a central antecedent of exploration-exploitation performance, which mediates the positive effect that emotional stability has on exploration-exploitation performance. Second, we found that emotional stability plays an additional and very important role in exploration-exploitation: this personality antecedent mediates the negative effect of recent, task-unrelated stress on cognitive flexibility. Thus, emotional stability acts as a protective shield by thwarting the detrimental effect of negative context signals on leaders’ cognition and, ultimately, on exploration-exploitation performance. Third, we found that present task motivation affects exploration-exploitation performance positively but indirectly through cognitive flexibility. This means that the motivation to conduct a certain task requiring exploration and exploitation favors the cognitive flexibility needed to show high performance in the corresponding decisions. Taken together, the results provide empirical evidence of leaders’ adaptive exploration-exploitation decisions taking place in a truly situated manner: they leverage cognitive flexibility and specific personality antecedents to process helpful and potentially harmful context-evoked signals to achieve higher exploration-exploitation performance.

We make two contributions to the understanding of the antecedents of exploration-exploitation performance in organizations. First, we contribute to the Carnegie literature by putting forward and testing an integrative model that studies together individual and context-evoked antecedents that predict exploration-exploitation performance. In contrast to reductionist approaches, our model and statistical approach lay the foundations for explaining the complex interplay between different mechanisms behind adaptive behavioral responses to exploration-exploitation problems. To do so, the model considers variables that capture fundamental antecedents of human decisions in everyday organizational life that affect each other and jointly affect exploration-exploitation. The individual antecedents considered are personality and cognitive flexibility, and the context-evoking antecedents rely on variables related to how individuals’ affective signals capture elements of the context over different time horizons. Some affective signals capture aspects of the recent past that are unrelated to the task environment. Our integrative approach shows that affective signals such as stress and task motivation can flaw and bias cognition or, on the contrary, capture important contextual cues, interact positively with cognition, and in turn lead to higher decision performance. We hope that this richer understanding of the antecedents of exploration-exploitation performance and their interactions in the setting of the Swiss Armed Forces can serve as the basis for future replications in other organizational settings.

Second, we contribute to psychology by putting context at the center of our model seeking to explain exploration-exploitation behavior. Organizational psychology suffers from a lack of research incorporating the role of context when explaining human behavior (Johns, 2018). The chosen lab-in-the-field approach allows us to combine the control of the lab with the realism of having participants respond to questions and tasks in their everyday setting over a long period of time—something that is fundamental when trying to capture context-evoked antecedents in a more realistic manner, favoring the external validity of our findings. Hence, our study provides further evidence for the notion that context and behavior are intrinsically linked through personality and cognition and that contextual factors are decisive for a better understanding of behavior in organizations. We shed further light on the role of perceived context by subdividing context-evoked antecedents according to their time horizon (recent past or present). Our findings show that recent, task-unrelated stress and present task motivation both influence behavior. However, present task-unrelated emotional states do not have an effect. This finding highlights the crucial role of motivation in activating cognitive flexibility and promoting vigilant switching between exploration and exploitation. Nevertheless, to make appropriate exploration-exploitation decisions in a periodically stressful environment, leaders must also overcome past pressures, focusing on the present and immediate priorities.

In addition, we hope that our study can serve as a basis for deriving practical implications. Our model presents antecedents that have a positive and negative impact on exploration-exploitation performance depending on contextual and individual conditions. While we do not claim that these findings are applicable to any organization, the rich picture that they paint of individual antecedents of adaptive exploration-exploitation behavior may guide organizations’ reflections toward improvements on both the individual and organizational levels. On the individual level, organizations could invest in the careful assessment of cognitive flexibility and emotional stability to select new leaders or promote existing leaders who already excel in these antecedents. Conversely, organizations can develop training programs for both their new and existing leaders to improve these antecedents through cognitive flexibility interventions (Buttelmann and Karbach, 2017). On the organizational level, it is possible to account for both the negative and positive effects of affective signals by implementing appropriate organizational designs and job roles that allow leaders to better cope with stressors and choose work tasks that motivate them. Furthermore, by cultivating the importance of self-awareness and emotional regulation, leaders can effectively prepare themselves for the critical moments of making exploration-exploitation decisions and transcend past pressures.

We are aware that the model we put forward presents only a limited representation of the myriad variables that make up a given context and might affect decision-making. In their writing, March and Olsen (1989, 1996) extended the notion of context to include broader social and cultural norms and values. We see at least four ways in which future studies could expand our efforts, which we outline below.

One, future studies could change the type of task to include more complex or even ill-structured tasks, which are vital for organizations (Baer et al., 2012). Most of the time, exploration-exploitation tasks are conceptualized and operationalized as well-structured tasks with predefined alternatives. However, exploration often involves not only choosing an unknown outcome but also coming up with an unknown alternative. Therefore, the antecedents we found in this study might not apply to ill-defined exploration-exploitation tasks. In contrast to our findings, or those of Laureiro-Martinez et al. (2019) and Bergenholtz et al. (2023) found no robust link between cognitive flexibility and exploration-exploitation performance. The reason for this finding might lie in the more complex tasks they used, to the point where cognitive flexibility could no longer play a positive role in performance. Clarifying this and identifying the boundary conditions for cognitive flexibility’s influence on exploration-exploitation performance could have useful theoretical and empirical implications.

Two, we see promise in expanding the variables that are part of the context and might affect exploration-exploitation performance, hopefully increasing the effect sizes in our model. We see the possibility of doing this in a very controlled manner, by manipulating the context, or in a less-controlled manner, by developing methods that would allow the consideration of more context variables while still capturing dynamic responses to exploration-exploitation problems. Both approaches have important advantages. Control over the amount of change in a certain context-evoked variable is promising in terms of deriving practical implications and could lead to causal results. Capturing dynamic exploration-exploitation decisions, meanwhile, is promising in terms of understanding the processes that unfold and the “values” of the variables with more realism, without the effect of artificial manipulations or rather extreme external shocks to the context. Work along these lines will open opportunities to study how interactions with other individuals shape interpretations of the context. The question is whether the personality and cognitive variables identified in our findings would continue to affect exploration-exploitation performance in significant ways if, for example, the decisions are taken jointly with other individuals.

Three, a potentially more psychologically oriented future development lies in further clarifying the role of emotional states as context-evoked antecedents. In our study, positive emotional states did not affect exploration-exploitation performance and we could not reliably measure the effect of negative emotional states due to the lack of variance. Correlations between positive emotional states and emotional stability let us speculate that emotional stability also captures the effect of (positive) emotional states on exploration-exploitation and that emotional states do not represent optimal antecedents to capture context.

However, this is rather speculative, and we suggest testing that assumption on another sample with more variance in both emotional states to solidify the relation. Further studies could advance our starting model to better understand the links between the variables themselves. As an example, let us take the positive correlation between positive emotional states and task motivation found in our study. Since it has been shown in previous studies that emotion can either enhance or impair cognitive performance, in order to have a better understanding of how emotional states affect cognitive flexibility and exploration-exploitation, we could consider an additional factor: the strength or arousal of the stimulus in relation to its task relevance. So, for example, when arousal is “high” and the stimulus/manipulation is task-irrelevant, resources are more fully diverted toward the processing of the emotional item and, because the mobilization of resources is more pronounced, the effects on behavior are greater (Mather and Sutherland, 2011). Future studies could manipulate or use more detailed measures and better understand how different levels of these variables affect each other.

Four, an empirical test of our microfoundational model in a non-military context would allow elaboration on whether our findings apply to leaders operating in less hierarchical and regulated organizations (e.g., startups) than the Swiss Armed Forces. Relatedly, and building on our argument that context indirectly affects performance through affective signals, we consider it important to understand whether the interplay between positive and negative context-evoked and individual antecedents would differently influence adaptive responses to the exploration-exploitation tension. In our study, gender, or being a female leader, showed a negative correlation with exploration-exploitation performance. However, the proportion of female leaders in our sample (20 out of 282) does not allow for the generalization of this finding. Given that remarkable gender imbalance, further studies are needed to study if gender does have an effect on adaptive exploration-exploitation decisions.

In conclusion, this study emphasizes the importance of the interplay between individual and context-evoked antecedents for adaptive exploration-exploitation decisions. Cognitive flexibility affects exploration-exploitation performance most directly by mediating the positive effects of emotional stability and context-evoked task motivation. Emotional stability, in turn, mediates the negative effect of context-evoked stress on cognitive flexibility. We interpret these mediation effects as evidence that emotionally stable leaders regulate the detrimental effect of recent context-evoked stress to facilitate the effective use of cognitive flexibility in a given exploration-exploitation task. Likewise, cognitive flexibility is further enhanced by the motivation to perform the task.

## Data availability statement

The datasets presented in this article are not readily available. This restriction is based on the approval of data use consented by the participants when they agreed to voluntarily participate in this study. Requests to access the datasets should be directed to DL-M, dlaureiro@ethz.ch.

## Ethics statement

The study was approved by Prof. Lutz Wingert ETH Zurich, Ethics Commission, and conducted in accordance with the local legislation and institutional requirements. The participants provided their written informed consent to participate in the study.

## Author contributions

ZZ-U and DL-M conceived the study. JR, ZZ-U, and DL-M designed the empirical strategy and wrote the manuscript. JR collected the data and performed the statistical analyses. All authors contributed to the manuscript revision and read and approved the submitted version.
